# Effects of Alpine land‐use changes: Soil macrofauna community revisited

**DOI:** 10.1002/ece3.3043

**Published:** 2017-06-12

**Authors:** Michael Steinwandter, Birgit C. Schlick‐Steiner, Gilg U. H. Seeber, Florian M. Steiner, Julia Seeber

**Affiliations:** ^1^ Institute of Ecology University of Innsbruck Innsbruck Austria; ^2^ Institute for Alpine Environment Eurac Research Bozen/Bolzano Italy; ^3^ Institute of Political Science University of Innsbruck Innsbruck Austria

**Keywords:** belowground diversity, dwarf shrub encroachment, earthworms, insect larvae, millipedes, semi‐natural grassland

## Abstract

Although soil invertebrates play a decisive role in maintaining ecosystem functioning, little is known about their structural composition in Alpine soils and how their abundances are affected by the currently ongoing land‐use changes. In this study, we re‐assessed the soil macrofauna community structure of managed and abandoned Alpine pastureland, which has already been evaluated 14 years earlier. Our results confirm clear shifts in the community composition after abandonment, in that (1) Chilopoda and Diplopoda were recorded almost exclusively on the abandoned sites, (2) Coleoptera larvae and Diptera larvae were more abundant on the abandoned than on the managed sites, whereas (3) Lumbricidae dominated on the managed sites. By revisiting managed and abandoned sites, we infer community patterns caused by abandonment such as changes in the epigeic earthworm community structure, and we discuss seasonal and sampling effects. Our case study improves the still limited understanding of spatio‐temporal biodiversity patterns of Alpine soil communities.

## 1 INTRODUCTION

Alpine ecosystems and, as part of these, Alpine soil communities are currently under socio‐economic pressure. For hundreds of years, high‐elevation grassland was shaped by traditional, extensive management and thus developed into species‐rich and stable, semi‐natural ecosystems (European Environment Agency, 2010; Niedrist, Tasser, Luth, Dalla Via, & Tappeiner, 2008). Alpine traditional and low‐intensity farming are characterized by holding small herds of livestock in stables in winter and driving them up on mountain pastures and meadows for grazing in summer. In addition, the grassland is mowed once every or every other year (Battaglini, Bovolenta, Gusmeroli, Salvador, & Sturaro, 2014). Benefits of these low‐intensity management practices include provisioning (e.g., food, fodder), regulating (e.g., natural hazard and water regulation, stabilization of slopes, Tasser, Walde, Tappeiner, Teutsch, & Noggler, 2007), and cultural services (e.g., tourism, esthetic, and recreation value, Lamarque et al., 2011). Consequently, semi‐natural high‐elevation grasslands are of highest conservation priority within Europe under the Habitat Directive 92/43/EEC (European Commission, 1992). However, in the recent past, managed Alpine areas have been increasingly abandoned. A loss of 20% of agricultural land over the entire Alps was recorded in the last century and of even 62% in the research area from 1954 to 2011 (Schirpke et al., 2013). The consequence, beside the loss of cultural heritage, increased soil erosion and surface runoff, as well as higher risks of avalanches and other natural hazards, is a decrease in the below‐ and aboveground biodiversity (Körner, Nakhutsrishvili, & Spehn, 2006; Tasser, Mader, & Tappeiner, 2003).

The vegetation of abandoned land undergoes several phases of what is secondary succession (Odum, 1969), which can have natural (e.g., landslides, Elias & Dias, 2009) or anthropogenic origins (e.g., land‐use changes, Rikhari, Negi, Ram, & Singh, 1993 and Bhaskar, Dawson, & Balvanera, 2014). In the most early stages of secondary succession, plant and animal biodiversity as well as primary production (i.e., biomass) are high, as new species colonizing the developing ecosystem join those from the earlier stage. As biotic and abiotic conditions change with time and become unfavorable for the former community, a new community establishes, and a shift in the species composition occurs (Connell & Slatyer, 1977; Kaufmann, 2001; Walker & del Moral, 2003). Depending on the given situation, secondary succession processes can take many years until the ecosystem and, in particular, the plant and animal community have reached stability again (Connell & Slatyer, 1977). Regretfully, ecologists have largely neglected belowground biodiversity and have focused mostly on aboveground species (Decaëns, 2010; Pärtel, Hiiesalu, Opik, & Wilson, 2012). Studies that have dealt with secondary successional patterns of the soil macrofauna community after land‐use changes reported consistent trends. Intensive agriculture has negative effects on the diversity and densities of soil invertebrates, as well as on the functioning of food chains, mainly due to the simplification of landscapes, soil degradation, and deterioration of water quality (Stoate et al., 2001; Ponge, Salmon, Benoist, & Geoffroy, 2015; but see Jedlička & Frouz, 2007). However, biodiversity may quickly recover once the disturbance is reduced (Ponge et al., 2015; Seeber et al., 2005), and farmland species might be replaced by wildlife species.

Shedding more light on soil species diversity present during and after secondary succession processes is crucial and could improve action plans to buffer and counteract ecosystem threats. Soil invertebrates play a decisive role in maintaining ecosystem functioning by mediating important ecosystem processes such as litter decomposition, nutrient cycling (especially of carbon and nitrogen), water infiltration, and nutrient storage in the soil (as summarized in Hagvar, 1998). Changes in the structural and functional composition of soil invertebrates due to land‐use abandonment, therefore, likely have considerable impacts on the function and stability of Alpine ecosystems (Bradford et al., 2014; Cardinale et al., 2012).

In this study, we revisited managed and abandoned Alpine grasslands that had already been investigated 14 years earlier (Seeber et al., 2005) with a statistically more sound study design. We have re‐addressed the question, how abandonment of management affects the structural composition of soil macrofauna communities, thereby providing first insights into the ecological dynamics of soil macrofauna communities in Alpine semi‐natural grassland following the cessation of traditional low‐intensity management.

## 2 MATERIAL AND METHODS

### 2.1 Ethics statement

The Institute of Ecology of the University of Innsbruck has a general permit to use the Kaserstattalm area for scientific purposes. No endangered or protected species were involved in the experiment.

### 2.2 Study site

Four sites, three semi‐natural and one intensively managed grassland investigated 14 years earlier, were resampled (see Seeber et al., 2005 and Fig. S1). They are part of the Alpine Long‐Term Ecological Research (LTER) area Kaserstattalm in the Stubai Valley (Central Alps, Tyrol, Austria, 47°07′N, 11°18′E). In detail, the four areas were as follows: (1) An intensively managed meadow (mM), fertilized and mowed once a year and grazed by cattle in late summer, located at 1,860 m above sea level (a.s.l.); (2) an abandoned and afforested meadow (aM), at 1,900 m a.s.l.; (3) a managed pasture (mP), grazed by cattle in summer, at 2,020 m a.s.l., and (4) an abandoned pasture (aP) at 2,000 m a.s.l. Details for the sites are summarized in Table 1. The cessation of management on both abandoned sites began in 1979, when an afforestation and slope‐amelioration program have been initiated to the close‐by unsecured river ditch (farmer Leo Pfurtscheller, pers. comm.). The affected areas were fenced, and since then no grazing cattle and sheep had access. From 1979 to 1988 several afforestation measurements with *Larix decidua* (L.), *Picea abies* (L.), and *Pinus cembra* (L.) were conducted; the area comprising aM was afforested in the mid‐1980s, while aP was not. All sites are located on south‐ to southeast exposed steep slopes (average of 25° inclination) on silicate bedrock, with influence of carbonate scree for the pastures due to erosion of surrounding peaks. The climate is central Alpine, continental, and temperate (Bitterlich & Cernusca, 1999) with a mean annual temperature of 2.4°C and a mean annual precipitation of 1,100 mm at 1,750 m a.s.l. (Schirpke et al., 2013).

**Table 1:** Soil properties of the 2012 samples. Soil parameters are given as the mean and standard deviation in parenthesis (*n* = 7)

Site	Plot code	Elevation [m a.s.l.]	Soil type	pH***	Soil organic matter [%]***	Concentration of carbon [%]***	Concentration of nitrogen [%]***	C/N ratio*
Managed meadow	mM	1,860	Eutric Cambisol	5.08 (0.39)^ac^	24.99 (4.40)^a^	7.80 (1.93)^a^	0.74 (0.16)^a^	10.57 (0.30)^a^
Abandoned and afforested meadow	aM	1,900	Dystric Cambisol	4.44 (1.17)^ab^	34.24 (7.59)^b^	8.27 (1.71)^a^	0.49 (0.10)^a^	18.26 (8.60)^b^
Managed pasture	mP	2,020	Dystric Cambisol	5.58 (0.27)^c^	17.37 (3.27)^ac^	13.35 (2.91)^b^	1.13 (0.25)^b^	12.78 (1.25)^ab^
Abandoned pasture	aP	2,000	Haplic Podzol	3.93 (0.04)^b^	17.07 (4.05)^c^	14.41 (3.49)^a^	0.64 (0.06)^a^	13.34 (1.48)^ab^

The results of the analysis of variance (with Tukey post hoc test), with sites as independent variable, are indicated with significance levels at *p* < .05 (*), *p* < .01 (**), and *p* < .001 (***).

Superscript letters indicate differences between the groups at *p* < .05 level.

### 2.3 Sampling

Soil core samples (diameter 30 cm, depth 15 cm, and seven replicates per site) were taken randomly at each site on 18 June and 17 July 2012, when Alpine vegetation production and soil community development were already well advanced into the season (Meyer & Thaler, 1995; Seeber et al., 2005). The distance between samples was at least 20 m. The samples were taken to the soil laboratory at the Institute of Ecology of the University of Innsbruck and processed within 4 hr. In the 1998 sampling, the same procedure has been applied with the exception that only 1–2 soil core samples have been taken per site, however, once a month between June and October 1998 (resulting in 8–10 samples per site).

The soil macrofauna community (invertebrates larger than 2 mm) was extracted by heat in a modified Kempson extractor (Kempson, Lloyd, & Ghelardi, 1963) for 12 days and collected in 75% ethylene glycol; samples were finally stored in 75% ethanol. The animals were determined to species (Lumbricidae, Diplopoda) or family (dipteran and coleopteran larvae, Chilopoda) level under a dissecting microscope. The identification of Lumbricidae followed Czusdi and Zicsi (2003), that of Diplopoda Pedroli‐Christen (1993) and Read (1990), and that of all other taxa Schaefer (2009). Biomasses were determined as fresh, dabbed‐dry weight corresponding to the taxonomic resolution used for abundances, that is, at the species/family level, using a microbalance (Sartorius, Göttingen, Germany) with an accuracy of 0.01 mg. Individuals belonging to the mesofauna community (<2 mm) and other taxa, which were not the aim of the study (e.g., Opiliones, Lepidoptera larvae, and Hymenoptera), were excluded. The soil was returned to the site of removal after the extraction of soil organisms to minimize destruction and fragmentation of the vulnerable Alpine pastureland.

### 2.4 Statistical analyzes

To assess the effects of site management on the abundance of species Generalized Linear Mixed Models (see, e.g., Bolker et al., 2009) were used. Due to the presence of overdispersion in the count data, these models use negative binomial distributions as their stochastic component. To account for correlated data induced by the sampling design, a random effect defined by the cross‐tabulation of site and (year of) sampling is included in all models. Calculations have been carried out with the open‐source statistical programming language R, version 3.3.2 (R Core Team, 2016) and the lme4‐package, version 1.1‐12 (Bates, Mächler, Bolker, & Walker, 2015). Models were calculated for main taxa Lumbricidae, Chilopoda, Diplopoda, Diptera larvae, Coleoptera, and Coleoptera larvae.

Differences in biomass within years were analyzed with a one‐way ANOVA with factor site (at significance level *p* < .05 and Tukey post hoc tests). Differences in biomass between years were analyzed with two‐way ANOVA with factors site and year (at significance level *p* < .05 and Tukey post hoc tests).

Calculations of biodiversity indices were based on the highest possible taxonomic resolution available for both datasets (1998 and 2012), that is Lumbricidae and Diplopoda at species level, Diptera larvae at family level, and Coleoptera, Coleoptera larvae, and Chilopoda at order level. Due to the coarse taxonomic resolution of the 1998 dataset, more elaborate calculations were not possible. Taxonomic richness (S, i.e., total number of species), the Shannon–Wiener Index (H’, Shannon, 1948), Simpson's Diversity Index (D, Simpson, 1949), and Pielou's Evenness (J’, Pielou, 1969) were calculated with the vegan‐package (Oksanen et al., 2016) in R for all sites to compare structural diversity for both years. Community composition and community–environment relationships were evaluated by principal component analysis and constrained correspondence analysis, respectively, using Canoco 5.04 (Ter Braak & Šmilauer, 2012).

Using the Bray–Curtis similarity index (Bray & Curtis, 1957), Analysis of Similarity (ANOSIM) of abundances was calculated to test whether the pairwise similarities of the communities within and between management (M, P), treatment (m, a) and, when applicable, year (1998, 2012) were the same. The significance of ANOSIM's statistic, R, was evaluated using 999 permutations and a threshold of α = 0.05. Following Lin et al. (2003), significant *R* ≥ .75 were interpreted as well separated, .50 ≤ *R* < .75 as separated but with a slight overlap, .25 ≤ *R* < .50 as separated but with a strong overlap, and *R* < .25 as hardly separable. In detail, ANOSIM was run for all samples of both years combined (*ALL*) and separately (1998, 2012) as well as for the four most abundant and functionally important (Meyer & Thaler, 1995; Seeber et al., 2005) Alpine soil invertebrate taxa, Lumbricidae, Diplopoda, Chilopoda, and Diptera larvae, of both years combined (*4Taxa*). Nonmetric Multidimensional Scaling ordination (NMDS) visualized the Bray‐Curtis dissimilarities (Clarke & Gorley, 2006). The Standardized Residual Sum of Squares (STRESS) was used to characterize the agreement of the original Bray‐Curtis values and the NMDS plot, with lower STRESS values indicating a better representation. In preliminary Bray‐Curtis calculations, we used various data transformations before calculations (untransformed, square root transformed, fourth‐root transformed, and presence/absence); in the final Bray‐Curtis calculations, we used untransformed and fourth‐root transformed data, because these two transformations had yielded the highest discriminative power in ANOSIM and NMDS. Fourth‐root transformation, Bray‐Curtis index, ANOSIM, NMDS, and STRESS were calculated via the PRIMER‐E software package v6.1.6 (Clarke & Gorley, 2006).

## 3 RESULTS

### 3.1 Abundances

We identified 3,420 individuals in 2012, belonging to 45 taxa, from a total of 56 soil samples. No clear numerical dominance of any taxon was found among the four sites. Generally, we found more individuals of Chilopoda, Diplopoda, Nematocera larvae, Brachycera larvae, and Coleoptera larvae in abandoned than in managed soils (Table 2). The 2012 taxa diversity was similar to that of 1998, with nearly the same taxa present (see Tables 2 and S1). However, the two sampling years differed markedly in the abundance of most main soil taxa, most numbers decreased.

**Table 2:** Descriptive mean abundances (individuals m^–2^) with standard deviation in parentheses of all heat extracted soil animals sampled in 2012

Abundance [ind. m^–2^]	Meadows		Pastures	
	Managed (mM)	Abandoned (aM)	Managed (mP)	Abandoned (aP)
Gastropoda (with shell)	11.12 (16.81)	33.35 (29.68)	9.09 (15.30)	13.14 (15.16)
Gastropoda (no shell)	2.02 (5.14)	3.03 (6.02)	3.03 (8.19)	1.01 (3.78)
Pseudoscorpiones	–	–	1.01 (3.78)	6.06 (16.38)
Araneae (Linyphiidae)	23.24 (29.68)	118.23 (88.19)	17.18 (22.32)	33.35 (39.86)
**Lumbricidae**	**152.59 (75.31)**	**147.53 (91.64)**	**115.20 (41.47)**	**117.22 (131.36)**
*Lumbricus rubellus*	55.58 (45.91)	50.53 (29.73)	53.56 (44.45)	35.37 (38.74)
*Dendrobaena octaedra*	46.48 (64.10)	31.33 (38.52)	33.35 (37.88)	70.74 (106.16)
*Octolasion lacteum*	25.26 (20.89)	12.13 (13.43)	8.08 (10.69)	1.01 (3.78)
*Allolobophora* sp.	25.26 (19.36)	53.56 (52.11)	20.21 (22.00)	10.11 (12.52)
**Chilopoda**	**–**	**204.12 (152.44)**	**12.13 (14.53)**	**197.05 (137.10)**
Lithobiidae	–	153.60 (128.69)	10.11 (12.93)	97.01 (97.52)
Geophilidae	–	50.53 (51.97)	2.02 (5.14)	100.04 (59.11)
**Diplopoda**	**3.03 (8.19)**	**168.75 (119.46)**	**63.66 (60.47)**	**132.38 (86.79)**
Crasposomatidae				
*Iulogona tirolensis*	–	–	1.01 (3.78)	1.01 (3.78)
Julidae	3.03 (8.19)	160.67 (114.74)	62.65 (60.46)	125.30 (83.76)
*Cylindroiulus fulviceps*	2.02 (7.56)	12.13 (27.10)	20.21 (24.64)	2.02 (7.56)
*Cylindroiulus meinerti*	1.01 (3.78)	28.29 (21.49)	14.15 (20.01)	72.76 (72.53)
*Enantiulus nanus*	–	120.25 (114.90)	28.29 (52.64)	50.53 (70.35)
Glomeridae				
*Glomeris hexasticha*	–	8.08 (10.69)	–	6.06 (7.27)
Heteroptera	7.07 (12.09)	5.05 (7.03)	17.18 (23.66)	5.05 (7.03)
Homoptera	57.60 (70.07)	94.99 (78.56)	180.88 (245.92)	65.68 (53.69)
**Diptera larvae**	**67.70 (105.60)**	**183.91 (125.31)**	**18.19 (21.08)**	**65.68 (41.00)**
**Nematocera larvae**	**49.51 (101.22)**	**102.06 (127.65)**	**14.15 (18.40)**	**33.35 (28.62)**
Chironomidae larvae	–	4.04 (8.65)	–	1.01 (3.78)
Bibionidae larvae	–	–	1.01 (3.78)	–
Cecidomyiidae larvae	26.27 (98.31)	49.51 (87.87)	7.07 (12.09)	17.18 (20.89)
Sciaridae larvae	10.11 (12.93)	21.22 (37.12)	5.05 (15.30)	13.14 (23.19)
Mycetophilidae larvae	–	7.07 (26.47)	–	–
Scatopsidae larvae	4.04 (11.68)	7.07 (13.31)	–	–
Tipulidae larvae	9.09 (13.14)	13.14 (41.33)	1.01 (3.78)	2.02 (5.14)
**Brachycera larvae**	**18.19 (15.12)**	**81.85 (52.40)**	**4.04 (8.65)**	**32.34 (33.95)**
Rhagionidae larvae	11.12 (14.87)	73.77 (50.00)	4.04 (8.65)	29.30 (34.41)
Empididae larvae	5.05 (8.96)	7.07 (12.09)	–	3.03 (6.02)
Anthomyiidae larvae	2.02 (5.14)	1.01 (3.78)	–	–
**Coleoptera**	**46.48 (41.68)**	**60.63 (33.49)**	**54.57 (35.47)**	**45.47 (30.98)**
Carabidae	11.12 (12.63)	5.05 (10.45)	22.23 (22.69)	2.02 (5.14)
Scarabaeidae	1.01 (3.78)	2.02 (7.56)	–	3.03 (8.19)
Staphylinidae	23.24 (26.38)	38.40 (31.11)	16.17 (18.28)	28.29 (29.88)
Pselaphidae	–	–	–	7.07 (15.45)
Silphidae	–	–	–	1.01 (3.78)
Histeridae	1.01 (3.78)	–	–	–
Elateridae	–	–	1.01 (3.78)	–
Anobiidae	–	–	1.01 (3.78)	–
Cryptophagidae	–	1.01 (3.78)	–	1.01 (3.78)
Chrysomelidae	4.04 (6.63)	8.08 (10.69)	1.01 (3.78)	1.01 (3.78)
Curculionidae	6.06 (9.14)	6.06 (10.69)	13.14 (19.59)	2.02 (5.14)
**Coleoptera larvae**	**112.17 (83.87)**	**248.58 (77.43)**	**139.45 (102.45)**	**103.07 (63.86)**
Carabidae larvae	26.27 (46.05)	44.46 (26.53)	5.05 (7.03)	21.22 (33.64)
Scarabaeidae larvae	10.11 (18.76)	–	2.02 (5.14)	1.01 (3.78)
Staphylinidae larvae	33.35 (27.53)	80.84 (54.49)	44.46 (47.69)	31.33 (31.48)
Elateridae larvae	6.06 (10.69)	61.64 (37.47)	41.43 (36.58)	5.05 (11.91)
Cantharidae larvae	8.08 (18.76)	56.59 (40.01)	8.08 (13.26)	31.33 (38.91)
Lampyridae larvae	–	–	1.01 (3.78)	–
Melyridae larvae	1.01 (3.78)	–	5.05 (7.03)	–
Coccinellidae larvae	2.02 (5.14)	–	1.01 (3.78)	–
Chrysomelidae larvae	15.16 (41.33)	3.03 (8.19)	3.03 (11.34)	–
Curculionidae larvae	10.11 (14.07)	2.02 (7.56)	28.29 (48.69)	13.14 (25.10)

Data presented for important taxa are bold, detailed identification of families and species are given where available.

Lumbricidae abundances in 2012 were lower on three of four sites compared with 1998 (mM, aM, aP); in both years more earthworms were found on meadows than on pastures (Tables 2, 3, and S1). The epigeic/hemiedaphic species *Lumbricus rubellus* (Hoffmeister, 1843) follows this general trend, while the endogeic species *Octolasion lacteum* (Orley, 1885) decreased in numbers on all four sites, especially on aP. The smaller epigeic species *Dendrobaena octaedra* (Savigny, 1826) increased in numbers on all sites except aM.

**Table 3:** Negative binomial generalized linear mixed models fit to abundance data

	Lumbricidae	Chilopoda	Diplopoda	Diptera larvae	Coleoptera	Coleoptera larvae
**Fixed effects**						
Intercept	2.476*** (0.088)	2.636*** (0.093)	2.169*** (0.305)	3.570*** (0.203)	1.675*** (0.172)	4.013*** (0.226)
Treatment	−0.364** (0.133)		0.682 (0.434)	0.122 (0.310)	−0.556* (0.261)	
Management		−3.600*** (0.279)	−3.543*** (0.587)	−0.779*** (0.212)		−1.785*** (0.324)
Sample				−1.099*** (0.214)		−1.552*** (0.291)
Treatment:management			2.215** (0.737)	−0.859* (0.356)		
Treatment:sample				−0.938**(0.349)		
Management:sample						1.504*** (0.417)
**Random effect**						
Site:sample (st.dev.)	<0.001	<0.001	0.344	<0.001	0.274	0.179
BIC	537.2	387.6	442.2	531.0	422.0	610.0
Deviance (*df*)	519.5 (79)	350.0 (79)	415.7 (77)	495.6 (75)	404.3 (79)	583.5 (77)
Number of observations	83	83	83	83	83	83
Number of groups	8	8	8	8	8	8

Significance codes: “***”: *p* < .001, “**”: *p* < .01, “*”: *p* < .05.

Indicator variables: treatment equals 1 for “pasture”, management equals 1 for “managed” and sample equals 1 for the 2012 sample. “:” denotes interaction of two effects.

Chilopoda were present almost exclusively on abandoned sites (Tables 2 and 3). Diplopoda were also more abundant on the abandoned sites (Table 3), but were found also in higher numbers on mP in both years (Table 2). *Cylindroiulus meinerti* (Verhoeff, 1891) greatly increased in numbers on aP from 1998 to 2012, while *Enantiulus nanus* (Latzel, 1884) showed the same increase on aM.

Larvae of Diptera and Coleoptera were more abundant on abandoned sites, however, their numbers greatly decreased on all sites from 1998 to 2012, except for a slight increase of Coleoptera larvae on mP (Tables 2, 3, and S1). Most notable are the lower densities of larvae of both taxa on aP in 2012, which can partly be ascribed to a strong decrease of more than 94% in the abundance of Cecidomyiidae larvae (Table 2). Adult Coleoptera were generally more abundant on meadows (Table 3), but showed similar abundances on all four sites in 2012 (Table 2).

**Figure 1:**
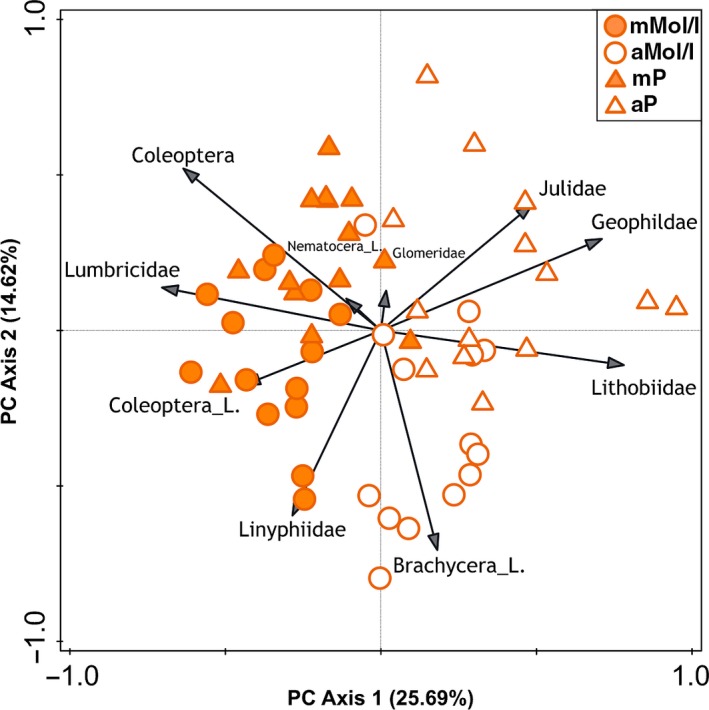
Unconstrained principal component analysis plot of log‐transformed abundance data (order/family level) for all 2012 samples for all four sites. Eigenvalues axis 1: 0.2569, axis 2: 0.1462, explained variation is 40.31%, total variation is 560.00

Managed and abandoned sites appear well separated on PC axis 1 in Figure  1, which summarizes the taxa (order/family level) distribution in 2012. Accordingly, primary decomposers (Lumbricidae), Nematocera larvae, and adult Coleoptera cluster on the left (managed) side, while predators (Lithobiidae, Geophilidae), Brachycera larvae, and Julidae are situated on the right (abandoned) side. A similar but more indistinct picture emerged for the 1998 taxa distribution (see Fig. S2). We also visualized the taxa distribution in 2012 using finer taxonomic resolution (Lumbricidae and Diplopoda at species level, all other taxa at family level) and obtained the same picture as at coarser resolution (Fig. S3).

### 3.2 Biomass

Generally, biomasses were low in 2012 and ranged from 6.56 to 8.98 g/m^2^. Mean Lumbricidae biomass was highest on all four sites in both years (Figs. S5 and S6) and contributed at least 60% to the overall biomass (except for the abandoned pasture in 2012: 37.19%). In contrast, lumbricid contributions to total abundance were much lower (7.88% on aP 1998 to 39.95% on mM in 2012).

### 3.3 Taxonomic diversity

Taxonomic richness decreased from 1998 to 2012 on three of four sites (mM, aM, and aP) and was higher on abandoned than on managed sites (except pastures in 2012). The Shannon–Wiener and Simpson's Diversity indices significantly differed between sites in 2012 (Table 4), the values were significantly higher for aM than for the other three sites. No significant differences were detected for Pielou's Evenness for both management type and year. All indices for 2012 were also calculated using the finer taxonomic resolution (family level for Chilopoda, Coleoptera, and Coleoptera larvae); ANOVA did not detect any significant differences in any index when comparing the taxonomic resolution levels (data not shown).

**Table 4:** Mean values for taxonomic richness, Shannon‐Wiener‐Index, Simpson's Diversity Index, and Pielou's Evenness for all four sites and the sampling years 1998 and 2012

Biodiversity	1998	2012 (low_res)	2012 (high_res)	1998 versus 2012 (low_res)
	Mean (*SD*)	Mean (*SD*)	Mean (*SD*)	
Taxonomic richness	*F*_3,23_ * *= 13.869, *p *< .001	*F*_3,52_ * *= 14.349, *p *< .001	*F*_3,52_ * *= 10.991, *p *< .001	*F*_3,75_ * *= 5.478, *p *=0.002
mM	12.89 (2.80)^a^	9.64 (1.82)^a^	11.79 (3.19)^a^	↓
aM	18.00 (2.39)^b^	14.57 (2.28)^b^	18.14 (3.18)^b^	↓
mP	10.60 (2.79)^a^	10.43 (1.70)^a^	13.00 (2.66)^a^	
aP	18.60 (2.07)^b^	11.64 (2.62)^a^	14.29 (3.36)^a^	↓
Shannon‐Wiener‐Index	*F*_3,23_ * *= 1.583, *p *= .221	*F*_3,52_ * *= 9.762, *p *< .001	*F*_3,52_ * *= 8.876, *p *< .001	*F*_3,75_ * *= 1.927, *p *= .132
mM	2.13 (0.27)	1.89 (0.22)^a^	2.10 (0.25)^a^	
aM	2.22 (0.36)	2.28 (0.11)^b^	2.56 (0.15)^b^	
mP	1.93 (0.29)	1.89 (0.28)^a^	2.13 (0.35)^a^	
aP	2.32 (0.24)	2.04 (0.24)^a^	2.30 (0.26)^ab^	
Simpson's Diversity Ind.	*F*_3,23_ * *= 0.551, *p *= .653	*F*_3,52_ * *= 4.693, *p *= .006	*F*_3,52_ * *= 4.241, *p *= .009	*F*_3,75_ * *= 1.815, *p *= .152
mM	0.84 (0.07)	0.80 (0.06)^a^	0.83 (0.05)^a^	
aM	0.82 (0.10)	0.87 (0.02)^b^	0.90 (0.02)^b^	
mP	0.80 (0.07)	0.78 (0.10)^a^	0.82 (0.11)^a^	
aP	0.86 (0.04)	0.83 (0.05)^ab^	0.87 (0.04)^ab^	
Pielou's Evenness	*F*_3,23_ * *= 0.931, *p *= .442	*F*_3,52_ * *= 0.993, *p *= .403	*F*_3,52_ * *= 1.213, *p *= .314	*F*_3,75_ * *= 1.628, *p *= .190
mM	0.84 (0.10)	0.84 (0.08)	0.87 (0.06)	
aM	0.77 (0.11)	0.86 (0.04)	0.89 (0.04)	
mP	0.83 (0.10)	0.81 (0.11)	0.84 (0.12)	
aP	0.79 (0.06)	0.84 (0.06)	0.87 (0.05)	

For the 2012 data, indices were calculated using once the low taxonomic resolution also available for 1998 (2012 low_res) and once the highest available taxonomic resolution (2012 high_res). *F*‐ and *p*‐values of Analysis of Variance for each index are shown in the respective first row.

Superscript letters indicate differences between the four sites at *p* < .05 level. The dataset for 1998 included 27 samples, the 2012 dataset 56 samples. SD, standard deviation.

Arrows indicate significant decrease of taxonomic richness between the two sample years.

### 3.4 Analysis of Similarity

**Figure 2:**
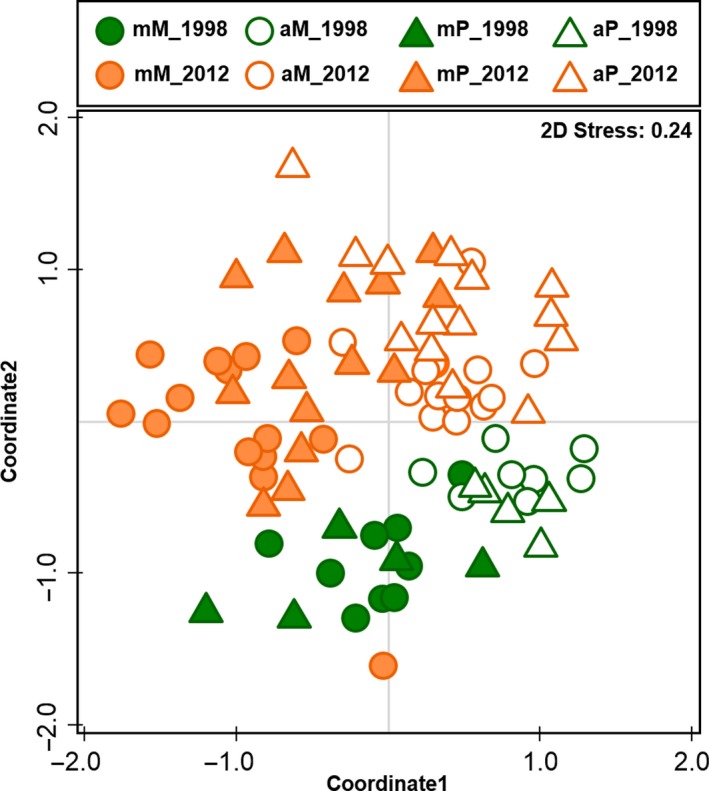
Nonmetric multidimensional scaling plot for all samples of both years combined after fourth‐root transformation. Management type (M, P), treatment (m, a), and year (1998, 2012) are indicated; for management type, treatment, and year, the *R* values were .116, .399, and .343, respectively (for details, see Table S4)

ANOSIM revealed the two management types analyzed, that is, meadow and pasture, as not separable, with *R* < .25 (Table S5) independently of which data were considered. The highest *R* values were returned for the treatment, that is, managed and abandoned, ranging from *R* = .388 (*ALL*, untransformed) to *R* = .581 (*4Taxa*, untransformed). Sampling year had likewise an effect (except for *4Taxa*, untransformed) but to a lesser degree; the effect increased from the untransformed to the fourth‐root transformed data. The example NMDS plot given in Figure 2 visualizes the mentioned effects quantified by ANOSIM for the *ALL* data after fourth‐root transformation, showing a separation with strong overlap with regard to years (orange – green) and treatment (filled and empty symbols) but no separation with regard to management (shape).

## 4 DISCUSSION

Inferences from the data presented are hampered by two problems arising from the 1998 study design. Firstly, replication of treatments is lacking and, secondly, the sites sampled are not truly independent as they are situated on the same mountain slope. Due to the scarceness of data on how Alpine soil communities change after management has been abandoned, there is still value to our case study, but generalizations beyond our case are not feasible because of these two pseudoreplication issues.

Before interpreting our findings, discussing the topic of taxonomic resolution is important. Using the term taxonomic sufficiency (Ellis, 1985), several studies have shown that the use of a coarse taxonomic resolution was enough to describe natural distribution patterns for various taxonomic groups (Fontaine, Devillers, Peres‐Neto, & Johnson, 2015), in limnic as well as terrestrial habitats (Riggins, Davis, & Hoback, 2009). Taxa for which a coarse taxonomy may work well include soil‐dwelling insect families, because in many instances, all members of a family perform the same or similar functions (e.g., carab beetle larvae are predators, sciarid larvae are fungal feeders). However, this might not be true for all taxa (for example ants, Riggins et al., 2009). Due to the limitations of the 1998 dataset, for most analyzes, we could use only few taxa at the species level (earthworms, millipedes) and had to use all others at the family (Diptera larvae) or order level (Coleoptera, Coleoptera larvae, and Chilopoda). We calculated diversity indices for the 2012 dataset once using the coarse taxonomic resolution necessary for the 1998 dataset and once using the finest taxonomic resolution possible and found no significant differences between the two analyzes (Table 4). Furthermore, principal component analyzes with both levels of resolution obtained similar results (Figure 1 and Fig. S3). These findings support the use of the coarse taxonomy in this study.

### 4.1 Soil community composition

In the temperate zone, earthworms (and, to a smaller extent, millipedes and soil‐dwelling Diptera larvae) act as ecological engineers by directly enhancing soil properties, aeration, and litter decomposition (Frouz, 1999; Lavelle et al., 1997). Because of the hundreds of years of extensive agricultural management of Alpine pastureland, a distinct soil macrofauna dominated by Lumbricidae has developed at areas near the timberline (Seeber et al., 2005). Our results at the order level showed clear shifts in the community composition after abandonment, in that (1) Chilopoda and Diplopoda were recorded almost exclusively on the abandoned sites (Figure 1 and Table 2), similar to situations found in montane forest soils (Meyer & Thaler, 1995), (2) Coleoptera larvae and Diptera larvae were more abundant in the abandoned than on the managed soils, whereas (3) Lumbricidae dominated on the managed sites.

Going into more detail, we found a separation (with a strong overlap) of communities with regard to both treatment and year (Figure 2 and Table S4). Taxa that immigrated soon after abandonment, that is were already present in 1998, mostly established well with high abundances. This applied particularly to millipedes, centipedes, and to some Brachycera families and Coleoptera larvae. The most abundant millipede species included in our study (*Cylindroiulus fulviceps* (Latzel, 1884)*, C. meinerti, Enantiulus nanus*) are characteristic woodland representatives (Pedroli‐Christen, 1993) and were present almost solely in the abandoned sites.

Within the earthworm community, we confirmed the presence of four Lumbricidae taxa on our research area (Seeber et al., 2005), all of which were present in varying numbers on each site. As cessation of management, a shift from the larger, epigeic/hemiedaphic *L. rubellus* to the smaller epigeic species *D. octaedra* took place, resulting in significantly lower earthworm biomasses in abandoned compared with managed areas. Although earthworm numbers decreased from 1998 to 2012, the increase in earthworm numbers on mP in 2012 might be explained with an extensification of management, as cattle and sheep were kept on the pasture for a shorter time during autumn and were driven finally on mM before returning to the valley (farmer Leo Pfurtscheller, pers. comm.).

The soil macrofauna community became more diverse between 1998 and 2012. This belowground biodiversity might strongly be linked to soil heterogeneity, which increases when abandonment proceeds (Pärtel et al., 2012). However, the retreat of earthworms with burrowing ability (*L. rubellus*) may have far‐reaching consequences for ecosystem functioning. The mixing of soil layers (especially the mixing of organic and mineral horizons) as well as amelioration processes such as aeration and water infiltration decline (Lavelle & Spain, 2001). *Dendrobaena octaedra* is not able to compensate the decomposition effort of *L. rubellus* (Seeber, Scheu, & Meyer, 2006), which is one reason, in addition to others such as increased amounts of recalcitrant shrub litter and lack of grazing, for the accumulation of soil organic matter on abandoned sites (e.g., the 2012 values for soil organic matter content were 34.24% ± 7.59 on aM compared with 24.99% ± 4.40 on mM, see Table 1 and Fig. S4). The result might be a shift from nutrient‐rich brown soils to more acidic podzols and distinct soil layers, which are more susceptible to erosion (Seeber & Seeber, 2005). This was confirmed, for example, by the measured pH of 5.58 ± 0.27 in mP compared with 3.93 ± 0.04 in aP (Table 1 and Fig. S4). At our research area Kaserstattalm, this podzolization was observed on a former pasture that had been abandoned 20 years earlier than our aP (Seeber & Seeber, 2005).

### 4.2 Community patterns

Despite their ecological importance, alpine areas are comparatively understudied (Nagy & Grabherr, 2009). To be better able to evaluate the effect of abandonment on the soil invertebrate community, we revisited sites that have been sampled 14 years prior to this study and confirmed most patterns found in 1998.

*Lumbricus rubellus* seems to prefer managed sites, while *D. octaedra* is more abundant on abandoned sites, the two, as a tendency, replacing each other. This general trend, discussed already by Seeber et al. (2005), can be confirmed with the 2012 data on the very pastures. On the abandoned meadow, however, *D. octaedra* significantly decreased in numbers, which might be caused by the development of an almost mature coniferous forest. Schwarz et al. (2015) reported significantly negative effects of *Larix decidua* on the earthworm community probably due to high C/N‐ratio and low litter palatability. The soil C/N‐ratio was significantly higher on aM compared with mM (18.26 ± 8.60 and 10.57 ± 0.30, respectively, Table 1). Acidic forest soils (Farley & Kelly, 2004; Ma, Zu, & Godron, 2001; Sterkenburg, Brandström‐Durling, Clemmensen, & Lindahl, 2015) are an unfavorable habitat for most earthworms species (pH < 4.5, Dominguez, 2004), explaining the overall decrease in earthworm numbers on this site in 2012. Likewise, Ponge et al. (2015) reported a shift from earthworms to macroarthropods from pastures via heathlands with different grazing intensities to pine forests. Similar findings on earthworm succession in Iceland after afforestation are also in line with our results (Sigurdsson & Gudleifsson, 2013).

Millipedes possibly immigrate after abandonment as trampling by grazing cattle ceases and dwarf shrubs emerge, providing them with a favorable habitat where they can find plenty of shelter and food beneath the shrubs and in the growing litter layer (David & Handa, 2010). David, Devernay, Loucougaray, and le Floc'h (1999) found higher millipede biodiversity in open shrubland (equivalent to our aP) and higher biodiversity and densities in mixed semi‐open sites (equivalent to our aM) compared to grazed open land (equivalent to our mP). This could explain the increase of millipedes from managed to abandoned sites in general and the large increase in numbers from 1998 to 2012 on aM. The decrease of millipedes on aP, however, might be a sampling effect: In 1998, samples were taken in a more ericaceous part of the site, but in 2012, we were forced to sample a more herbaceous part, as a permanent climate station had by then been built on the spot sampled in 1998.

Interpreting our findings on insect larvae is a bit more challenging. Frouz (1997) studied soil‐dwelling dipteran larvae in abandoned fields and showed clear succession patterns: Shortly after abandonment, humus feeders such as chironomid larvae dominate, while in later successional stages, mycetophagous, and saprophagous larvae such as Cecidomyiidae and Sciaridae increase in numbers. All three dipteran families were present in our 1998 dataset but were almost missing in 2012. However, when looking at the raw data of 1998, when sampling spanned from June to October, a clear seasonal effect is notable; most dipteran families occur in high numbers only in late summer and early autumn. As the samples in 2012 were taken in early and midsummer, we did not catch later emerging species. We found high numbers of dipteran larvae in 1998 in abandoned fields, confirming findings of Frouz (1997), who related the immediate increase in abundances after the cessation of land‐use to the dense vegetation and the thicker humus layer on these areas. However, lacking data of late summer and early autumn in 2012, we cannot evaluate that aspect of the successional pattern.

An additional potential cause for differences in abundances are annual fluctuations due to weather variations, which can have considerable influence on the development of, among others, invertebrate larvae hibernating in the soil (Frouz, 1999; Meyer & Thaler, 1995). While the winter of 1997/1998 was mild with average precipitation and snowfall, the winter of 2011/2012 was the warmest in Austrian mountain areas since the start of temperature records in 1851 (The Central Institution for Meteorology and Geodynamics, Austria, ZAMG). These dynamics likely further influenced the detected abundances of most soil‐dwelling Diptera and Coleoptera larvae that hibernate beneath the snow cover and hatch soon after snow melting at the beginning of the growing season.

## 5 CONCLUSIONS

In our study, we revisited managed and abandoned alpine pastureland to evaluate the effect of abandonment of land‐use on the soil macrofauna community structure. Our results show that the community becomes more diverse, which in itself is a positive effect. However, future studies should now focus on investigating (1) whether this is also true on the functional level and subsequently (2) what impact the new community structure has on ecosystem functioning.

## CONFLICT OF INTEREST

None declared.
