# Supplementary material for: Effects of Alpine land‐use changes: Soil macrofauna community revisited
Source: Ecol Evol. 2017 Jun 12;7(14):5389–99. doi: 10.1002/ece3.3043 (PMC5528212; doi:10.1002/ece3.3043)
Supplement: Supplementary file 1 [file ECE3-7-5389-s001.docx]

**Supporting Information for the Manuscript:**

**Effects of Alpine land-use changes: soil macrofauna community revisited**

Michael Steinwandter^1,2,^*, Birgit C. Schlick-Steiner^1^, Gilg U.H. Seeber^3^, Florian M. Steiner^1^, Julia Seeber^1,2^

^1^ Institute of Ecology, University of Innsbruck, Technikerstrasse 25, 6020 Innsbruck, Austria

^2^ Institute for Alpine Environment, Eeurac Research, Viale Druso 1, 39100 Bozen/Bolzano, Italy

^3^ Department of Political Science, University of Innsbruck, Universitätsstrasse 15, 6020 Innsbruck, Austria

^*^ Corresponding author:

Email: [Michael.Steinwandter@eurac.edu](mailto:Michael.Steinwandter@eurac.edu)

**Table S1: Mean abundances (individuals m^‒2^) with standard deviation in parentheses of all heat extracted soil animals sampled in 1998.** Data presented in bold for important taxa; detailed identification of families and species are given where available. The results of the analysis of variance (with Tukey post-hoc test), with ‘sites’ as independent variable, are indicated for all groups with significance levels at *P* < 0.05 (*), *P* < 0.01 (**), and *P* < 0.001 (***). Superscript letters indicate differences between the groups at *P* < 0.05 level.
N.B.: Indeterminable Diptera larvae were pooled as ‘Diptera larvae indet.’

| ABUNDANCE | MEADOWS | | | | | | PASTURES | | | | | | | *P*‒value |
| --- | --- | --- | --- | --- | --- | --- | --- | --- | --- | --- | --- | --- | --- | --- |
| [ind. m^‒2^] | **managed (mM)** | | | **abandoned (aM)** | | | **managed (mP)** | | | | **abandones (aP)** | | |  |
| Gastropoda (with shell) | 39.30 | (51.39) ^oo^ | 102.57 | | (78.86) ^oo^ |  | 2.83 | | (6.33) ^oo^ | 161.28 | | (190.22) ^oo^ |  | 0.054 |
| Gastropoda (no shell) * | ‒ | ^ao^ | 5.31 | | (7.32)  ^ao^ |  | ‒ | | ^ao^ | ‒ | | ^ao^ |  | 0.041 |
| Pseudoscorpiones * | ‒ | ^ao^ | ‒ | | ^ao^ |  | ‒ | | ^ab^ | 14.15 | | (20.01) ^bo^ |  | 0.022 |
| Araneae (Linyphiidae) | 130.47 | (104.15) ^oo^ | 173.30 | | (101.10) ^oo^ |  | 36.78 | | (39.51) ^oo^ | 147.13 | | (85.24)^oo^ |  | 0.097 |
| Lumbricidae * | **191.77** | **(68.62)** ^ao^ | **205.13** | | **(70.53)** ^ao^ |  | **59.42** | | **(27.21)** ^bo^ | **178.25** | | **(110.31)** ^ab^ |  | **0.011** |
| *Lumbricus rubellus* ** | 105.32 | (36.83) ^ao^ | 56.59 | | (27.26) ^bo^ |  | 42.44 | | (26.47) ^bo^ | 53.76 | | (20.98) ^bo^ |  | 0.002 |
| *Dendrobaena octaedra* *** | 14.15 | (22.37) ^ao^ | 123.79 | | (34.45) ^bo^ |  | 2.83 | | (6.33) ^ao^ | 45.27 | | (50.42) ^ao^ |  | < 0.001 |
| *Octolasion lacteum* | 40.87 | (34.25) ^oo^ | 19.45 | | (25.01) ^oo^ |  | 8.49 | | (7.75) ^oo^ | 45.27 | | (50.42) ^oo^ |  | 0.191 |
| *Allolobophora* sp. * | 31.44 | (26.26) ^ao^ | 5.31 | | (10.53) ^ao^ |  | 5.66 | | (7.75) ^ao^ | 33.95 | | (25.70) ^ao^ |  | 0.018 |
| Chilopoda *** | **6.29** | **(12.48)** ^ao^ | **183.91** | | **(83.18)** ^bo^ |  | **‒** | | ^ao^ | **200.89** | | **(61.18)** ^bo^ |  | **< 0.001** |
| Diplopoda *** | **4.72** | **(14.15)** ^ao^ | **83.11** | | **(82.64)** ^ao^ |  | **67.91** | | **(64.37)** ^ao^ | **540.42** | | **(202.65)** ^bo^ |  | **< 0.001** |
| Julidae *** | 4.72 | (14.15) ^ao^ | 67.20 | | (53.34) ^ao^ |  | 67.91 | | (64.37) ^ao^ | 540.42 | | (202.65) ^bo^ |  | < 0.001 |
| *Cylindroiulus fulviceps* *** | ‒ | ^ao^ | ‒ | | ^ao^ |  | 8.49 | | (18.98) ^ao^ | 56.59 | | (24.50) ^bo^ |  | < 0.001 |
| *Cylindroiulus meinerti* ** | ‒ | ^ao^ | 35.37 | | (31.18) ^bo^ |  | 8.49 | | (12.65) ^ab^ | ‒ | | ^ao^ |  | 0.002 |
| *Enantiulus nanus* *** | 3.14 | (9.43) ^ao^ | 31.83 | | (45.21) ^ao^ |  | 48.10 | | (71.86) ^ao^ | 483.83 | | (184.02) ^bo^ |  | < 0.001 |
| Glomeridae |  |  |  | |  |  |  | |  |  | |  |  |  |
| *Glomeris hexasticha* | ‒ |  | 15.92 | | (39.61) ^oo^ |  | ‒ | |  | ‒ | |  |  | 0.413 |
| Heteroptera ^n.s.^ | 3.14 | (6.24) ^oo^ | 3.54 | | (6.55) ^oo^ |  | 22.64 | | (29.34) ^oo^ | 2.83 | | (6.33) ^oo^ |  | 0.063 |
| Homoptera ^n.s.^ | 293.95 | (344.05) ^oo^ | 311.24 | | (209.70) ^oo^ |  | 178.25 | | (153.22) ^oo^ | 316.90 | | (276.25) ^oo^ |  | 0.814 |
| Diptera larvae * | **264.08** | **(147.53)** ^ao^ | **426.18** | | **(335.33)** ^ao^ |  | | **79.22** | **(55.34)** ^ab^ | **696.04** | | **(502.71)** ^ac^ |  | **0.017** |
| Nematocera larvae | **166.62** | **(118.32)** ^ao^ | **231.66** | | **(206.67)** ^ao^ |  | | **36.78** | **(16.13)** ^ao^ | **500.81** | | **(521.49)** ^ao^ |  | **0.051** |
| Ceratopogonidae larvae | 4.72 | (14.15) ^oo^ | 12.38 | | (15.93) ^oo^ |  | | ‒ |  | 22.64 | | (50.61) ^oo^ |  | 0.460 |
| Chironomidae larvae | 29.87 | (29.54) ^oo^ | 70.74 | | (116.41) ^oo^ |  | | 5.66 | (7.75) ^oo^ | 141.47 | | (196.03) ^oo^ |  | 0.193 |
| Cecidomyiidae larvae * | 121.04 | (91.71) ^ao^ | 81.35 | | (77.02) ^ao^ |  | | 28.29 | (17.33) ^ab^ | 291.43 | | (274.07) ^ac^ |  | 0.025 |
| Sciaridae larvae | 4.72 | (7.07) ^oo^ | 7.07 | | (10.69) ^oo^ |  | | 2.83 | (6.33) ^oo^ | 19.81 | | (29.34) ^oo^ |  | 0.243 |
| Mycetophilidae larvae | ‒ |  | 3.54 | | (6.55) ^oo^ |  | | ‒ |  | 2.83 | | (6.33) ^oo^ |  | 0.324 |
| Scatopsidae larvae | ‒ |  | 14.15 | | (34.65) ^oo^ |  | | ‒ |  | 16.98 | | (37.96) ^oo^ |  | 0.530 |
| Tipulidae larvae | 6.29 | (10.28) ^oo^ | 40.67 | | (61.17) ^oo^ |  | | ‒ |  | 5.66 | | (7.75) ^oo^ |  | 0.126 |
| Limoniidae larvae | ‒ |  | 1.77 | | (5.00) ^oo^ |  | | ‒ |  | ‒ | |  |  | 0.522 |
| Brachycera larvae * | **50.30** | **(46.98)** ^ao^ | **123.79** | | **(90.66)** ^ao^ |  | | **28.29** | **(26.47)** ^ao^ | **113.18** | | **(67.85)** ^ao^ |  | **0.037** |
| Rhagionidae larvae | 11.00 | (28.10) ^oo^ | 61.89 | | (73.68) ^oo^ |  | | 2.83 | (6.33) ^oo^ | 28.29 | | (17.33) ^oo^ |  | 0.084 |
| Asilidae larvae | 1.57 | (4.72) ^oo^ | ‒ | |  |  | | 2.83 | (6.33) ^oo^ | ‒ | |  |  | 0.539 |
| Empididae larvae | 23.58 | (25.50) ^oo^ | 42.44 | | (37.81) ^oo^ |  | | 11.32 | (11.84) ^oo^ | 56.59 | | (45.84) ^oo^ |  | 0.127 |
| Syrphidae larvae | ‒ |  | 1.77 | | (5.00) ^oo^ |  | | ‒ |  | ‒ | |  |  | 0.522 |
| Muscidae larvae | 14.15 | (14.15) ^oo^ | 17.68 | | (18.13) ^oo^ |  | | 11.32 | (18.45) ^oo^ | 28.29 | | (26.47) ^oo^ |  | 0.857 |
| Diptera larvae indet. | 47.16 | (38.09) ^oo^ | 70.74 | | (71.74) ^oo^ |  | | 14.15 | (24.50) ^oo^ | 82.05 | | (95.64) ^oo^ |  | 0.303 |
| Coleoptera * | **91.17** | **(53.46)** ^ao^ | **134.40** | | **(98.60)** ^ab^ |  | | **19.81** | **(21.46)** ^ac^ | **50.93** | | **(30.99)** ^ao^ |  | **0.026** |
| Coleoptera larvae * | **138.33** | **(123.29)** ^ao^ | **930.17** | | **(816.33)** ^bo^ |  | | **121.67** | **(70.45)** ^ab^ | **599.84** | | **(620.43)** ^ab^ |  | **0.019** |

**Table S2: Mean biomasses (mg fresh weight m^‒2^) with standard deviation in parentheses of all heat extracted soil animals sampled in 2012.** Data presented in bold for important taxa; detailed identification of families and species are given where available. The results of the analysis of variance (with Tukey post-hoc test), with site as independent variable, are indicated for the main groups with significance levels at *P* < 0.05 (*), *P* < 0.01 (**), and *P* < 0.001 (***). Superscript letters indicate differences between the groups at *P* < 0.05 level.

| BIOMASS | MEADOWS | | | | | | PASTURES | | | | | | *P*‒value |
| --- | --- | --- | --- | --- | --- | --- | --- | --- | --- | --- | --- | --- | --- |
| [mg fresh weight m^‒2^] | **managed (mM)** | | | **abandoned (aM)** | | | **Pasture (mP)** | | | **Pasture (aP)** | | |  |
| Lumbricidae | **6324.06** | **(3784.19)** ^oo^ |  | **5378.38** | **(4276.73)** ^oo^ |  | **5458.77** | **(3951.28)** ^oo^ | **2436.16** | | **(3158.23)** ^oo^ |  | **0.051** |
| *Lumbricus rubellus* | 3283.44 | (2053.09) ^oo^ |  | 2418.09 | (3229.07) ^oo^ |  | 3712.84 | (3727.56) ^oo^ | 1280.22 | | (2650.17) ^oo^ |  | 0.147 |
| *Dendrobaena octaedra* | 726.55 | (979.79) ^oo^ |  | 463.32 | (636.13) ^oo^ |  | 516.60 | (919.56) ^oo^ | 851.05 | | (1449.47) ^oo^ |  | 0.735 |
| *Octolasion lacteum ** | 1714.93 | (1788.90) ^ao^ |  | 1496.66 | (1942.00) ^ab^ |  | 692.43 | (1214.97) ^ab^ | 59.01 | | (220.81) ^ba^ |  | 0.015 |
| *Allolobophora* sp. * | 599.13 | (656.35) ^ao^ |  | 1000.30 | (704.42) ^ab^ |  | 536.90 | (676.24) ^aa^ | 247.88 | | (393.13) ^ac^ |  | 0.022 |
| Chilopoda *** | **‒** | ^ao^ | **302.62** | | **(277.64)** ^bo^ |  | **29.21** | **29.21** | **344.92** | | **(324.24)** ^bo^ |  | **< 0.001** |
| Lithobiidae ** | ‒ | ^ao^ | 198.34 | | (190.32) ^bc^ |  | 26.82 | 26.82 | 237.24 | | (395.00) ^co^ |  | 0.001 |
| Geophilidae ** | ‒ | ^ao^ | 104.27 | | (153.34) ^bo^ |  | 2.39 | 2.39 | 107.68 | | (82.09) ^bo^ |  | 0.001 |
| Diplopoda *** | **24.28** | **(88.52)** ^ao^ | **1829.69** | | **(1313.76)** ^bo^ |  | **1016.54** | **1016.54** | **2352.90** | | **(2178.90)** ^bo^ |  | **< 0.001** |
| Crasposomatidae |  |  |  | |  |  |  |  |  | |  |  |  |
| *Iulogona tirolensis* | ‒ |  | ‒ | |  |  | 2.67 | 2.67 | 1.27 | | (4.76) ^oo^ |  | 0.402 |
| Julidae ** | 24.28 | (88.25) ^ao^ | 1452.11 | | (926.82) ^bo^ |  | 1013.87 | 1013.87 | 2026.33 | | (2195.38) ^bo^ |  | 0.002 |
| *Cylindroiulus fulviceps* *** | 0.59 | (2.19) ^ab^ | 81.54 | | (191.34) ^bo^ |  | 773.63 | 773.63 | 1.79 | | (6.69) ^bo^ |  | < 0.001 |
| *Cylindroiulus meinerti* *** | 23.70 | (88.66) ^ao^ | 809.54 | | (811.59) ^ab^ |  | 79.99 | 79.99 | 1866.73 | | (2282.00) ^bo^ |  | < 0.001 |
| *Enantiulus nanus* *** | ‒ | ^ao^ | 561.03 | | (488.78) ^bo^ |  | 160.25 | 160.25 | 157.81 | | (303.67) ^ao^ |  | < 0.001 |
| Glomeridae |  |  |  | |  |  |  |  |  | |  |  |  |
| *Glomeris hexasticha* * | ‒ | ^o ao^ | 377.58 | | (683.01) ^ao^ |  | ‒ | ^ao^ | 325.29 | | (506.08) ^ao^ |  | 0.030 |
| Diptera larvae | **531.65** | **(570.98)** ^oo^ | **471.81** | | **(713.86)** ^oo^ |  | **20.69** | **20.69** | **335.72** | | **(602.45)** ^oo^ |  | **0.076** |
| Nematocera larvae | **427.10** | **(575.81)** ^oo^ | **196.07** | | **(466.51)** ^oo^ |  | **10.00** | **10.00** | **252.11** | | **(618.82)** ^oo^ |  | **0.164** |
| Chironomidae larvae | ‒ |  | 7.81 | | (28.34) ^oo^ |  | ‒ | ‒ | 0.23 | | (0.87) ^oo^ |  | 0.382 |
| Bibionidae larvae | ‒ |  | ‒ | |  |  | 0.38 | 0.38 | ‒ | |  |  | 0.400 |
| Cecidomyiidae larvae | 0.83 | (3.10) ^oo^ | 5.46 | | (7.45) ^oo^ |  | 2.92 | 2.92 | 1.85 | | (2.86) ^oo^ |  | 0.165 |
| Sciaridae larvae | 5.22 | (13.33) ^oo^ | 1.17 | | (2.57) ^oo^ |  | 6.52 | 6.52 | 7.02 | | (19.80) ^oo^ |  | 0.795 |
| Mycetophilidae larvae | ‒ |  | 0.28 | | (1.06) ^oo^ |  | ‒ | ‒ | ‒ | |  |  | 0.400 |
| Scatopsidae larvae | 4.22 | (12.75) ^oo^ | 3.87 | | (6.85) ^oo^ |  | ‒ | ‒ | ‒ | |  |  | 0.235 |
| Tipulidae larvae | 416.82 | (155.53) ^oo^ | 177.48 | | (470.79) ^oo^ |  | 0.18 | 0.18 | 243.01 | | (622.35) ^oo^ |  | 0.168 |
| Brachycera larvae | **104.55** | **(204.27)** ^oo^ | **275.74** | | **(554.09)** ^oo^ |  | **10.68** | **10.68** | **83.61** | | **(85.05)** ^oo^ |  | **0.129** |
| Rhagionidae larvae | 46.55 | (150.97) ^oo^ | 113.09 | | (124.28) ^oo^ |  | 10.68 | 10.68 | 80.13 | | (83.15) ^oo^ |  | 0.156 |
| Empididae larvae | 56.63 | (157.57) ^oo^ | 159.97 | | (573.35) ^oo^ |  | ‒ | ‒ | 3.48 | | (7.29) ^oo^ |  | 0.373 |
| Anthomyiidae larvae | 1.36 | (4.79) ^oo^ | 2.68 | | (10.02) ^oo^ |  | ‒ | ‒ | ‒ | |  |  | 0.400 |
| Coleoptera | **384.47** | **(734.64)** ^oo^ | **324.18** | | **(260.21)** ^oo^ |  | **247.79** | **247.79** | **588.04** | | **(961.88)** ^oo^ |  | **0.567** |
| Carabidae | 34.27 | (101.38) ^oo^ | 97.42 | | (190.45) ^oo^ |  | 47.72 | (113.55) ^oo^ | 61.88 | | (151.10) ^oo^ |  | 0.676 |
| Scarabaeidae | 69.88 | (261.45) ^oo^ | 12.72 | | (47.60) ^oo^ |  | ‒ |  | 54.88 | | (146.83) ^oo^ |  | 0.572 |
| Staphylinidae | 100.74 | (148.98) ^oo^ | 150.90 | | (137.64) ^oo^ |  | 28.07 | (42.65) ^oo^ | 109.06 | | (166.18) ^oo^ |  | 0.116 |
| Pselaphidae ** | ‒ | ^ao^ | ‒ | | ^ao^ |  | ‒ | ^ao^ | 1.54 | | (2.64) ^bo^ |  | 0.005 |
| Silphidae | ‒ |  | ‒ | |  |  | ‒ |  | 245.57 | | (922.60) ^oo^ |  | 0.258 |
| Histeridae | 31.72 | (118.68) ^oo^ | ‒ | |  |  | ‒ |  | ‒ | |  |  | 0.400 |
| Elateridae | ‒ |  | ‒ | |  |  | 121.40 | (454.25) ^oo^ | ‒ | |  |  | 0.400 |
| Chrysomelidae | 110.89 | (323.20) ^oo^ | 8.44 | | (13.25) ^oo^ |  | 0.27 | (1.02) ^oo^ | 75.54 | | (282.63) ^oo^ |  | 0.465 |
| Curculionidae | 36.97 | (73.54) ^oo^ | 54.70 | | (101.47) ^oo^ |  | 50.21 | (68.30) ^oo^ | 17.50 | | (46.64) ^oo^ |  | 0.561 |
| Coleoptera larvae * | **572.36** | **(893.46)** ^ao^ | **676.61** | | **(611.70)** ^ao^ |  | **1734.91** | **(1918.10)** ^ao^ | **496.79** | | **(1165.72)** ^ao^ |  | **0.037** |
| Carabidae larvae | 108.48 | (303.88) ^oo^ | 28.93 | | (28.44) ^oo^ |  | 16.68 | (57.16) ^oo^ | 154.85 | | (548.55) ^oo^ |  | 0.611 |
| Scarabaeidae larvae | 27.73 | (86.98) ^oo^ | ‒ | |  |  | 3.60 | (12.82) ^oo^ | 10.20 | | (28.64) ^oo^ |  | 0.404 |
| Staphylinidae larvae | 28.05 | (51.15) ^oo^ | 21.84 | | (20.67) ^oo^ |  | 85.72 | (207.60) ^oo^ | 30.17 | | (58.35) ^oo^ |  | 0.401 |
| Elateridae larvae ** | 363.10 | (624.85) ^ao^ | 590.83 | | (594.07) ^ab^ |  | 1580.29 | (1967.36) ^bo^ | 127.17 | | (378.00) ^ao^ |  | 0.005 |
| Cantharidae larvae | 9.59 | (24.78) ^oo^ | 26.85 | | (37.82) ^oo^ |  | 6.09 | (13.84) ^oo^ | 161.42 | | (515.49) ^oo^ |  | 0.338 |
| Melyridae larvae | 0.42 | (1.59) ^oo^ | ‒ | |  |  | 8.04 | (18.48) ^oo^ | ‒ | |  |  | 0.066 |
| Coccinellidae larvae | 12.97 | (45.85) ^oo^ | ‒ | |  |  | 0.17 | (0.64) ^oo^ | ‒ | |  |  | 0.353 |
| Chrysomelidae larvae | 5.72 | (11.36) ^oo^ | 7.65 | | (24.94) ^oo^ |  | 7.94 | (21.63) ^oo^ | ‒ | |  |  | 0.603 |
| Curculionidae larvae | 16.30 | (28.90) ^oo^ | 0.30 | | (1.13) ^oo^ |  | 26.36 | (53.10) ^oo^ | 12.97 | | (27.99) ^oo^ |  | 0.237 |

**Table S3: Mean biomasses with standard deviation in parentheses (mg fresh weight m^‒2^) of all heat extracted soil animals sampled in 1998.** Data presented in bold for important taxa; detailed identification of families and species are given where available. The results of the analysis of variance (with Tukey post-hoc test), with sites as independent variable, are indicated for the main groups with significance levels at *P* < 0.05 (*), *P*< 0.01 (**), and *P* < 0.001 (***). Superscript letters indicate differences between the groups at *P* < 0.05 level.
N.B.: indeterminable Diptera larvae were pooled as ‘Diptera larvae indet.’.

| BIOMASS | MEADOWS | | | | | | PASTURES | | | | | *P*‒value |
| --- | --- | --- | --- | --- | --- | --- | --- | --- | --- | --- | --- | --- |
| [mg fresh wt m^‒2^] | **managed (mM)** | | | **abandoned (aM)** | | | **managed (mP)** | | | **abandoned (aP)** | |  |
| Gastropoda (shell) ** | 265.81 | (382.11) ^ao^ | 256.06 | | (194.55) ^ao^ |  | 15.28 | (34.16) ^ao^ | 1308.89 | | (902.78) ^bo^ | 0.001 |
| Gastropoda (no shell) | ‒ |  | 176.49 | | (345.48) ^oo^ |  | ‒ |  | ‒ | |  | 0.215 |
| Pseudoscorpiones * | ‒ | ^ao^ | ‒ | | ^ab^ |  | ‒ | ^ab^ | 8.49 | | (13.08) ^bo^ | 0.039 |
| Araneae | 81.42 | (92.28) ^oo^ | 238.38 | | (318.66) ^oo^ |  | 20.65 | (32.19) ^oo^ | 210.23 | | (272.68) ^oo^ | 0.255 |
| Lumbricidae | **30432.00** | **(18488.34)** ^oo^ | **15970.31** | | **(10360.16)** ^oo^ |  | **16806.76** | **(16031.36)** ^oo^ | **15892.86** | | **(11261.51)** ^oo^ | **0.163** |
| *Lumbricus rubellus* | 22934.03 | (15559.80) ^oo^ | 10187.68 | | (7298.47) ^oo^ |  | 15759.88 | (16362.58) ^oo^ | 8847.60 | | (10412.22) ^oo^ | 0.159 |
| *Dendrobaena octaedra* *** | 565.88 | (1098.11) ^ao^ | 3996.56 | | (2200.45) ^bo^ |  | 84.88 | (189.80) ^ao^ | 962.00 | | (1102.20) ^ao^ | < 0.001 |
| *Octolasion lacteum* | 3363.87 | (4231.01) ^oo^ | 1662.28 | | (2487.12) ^oo^ |  | 254.65 | (253.07) ^oo^ | 3225.54 | | (5612.14) ^oo^ | 0.435 |
| *Allolobophora* sp. | 1996.31 | (2428.24) ^oo^ | 123.79 | | (232.31) ^oo^ |  | 707.36 | (1503.86) ^oo^ | 2857.72 | | (2127.72) ^oo^ | 0.051 |
| Chilopoda *** | **23.74** | **(67.07)** ^ao^ | **844.94** | | **(601.37)** ^bo^ |  | **‒** | ^ao^ | **290.30** | | **(264.29)** ^ab^ | **< 0.001** |
| Diplopoda ** | **242.86** | **(728.58)** ^ao^ | **1060.15** | | **(1216.11)** ^ao^ |  | **1202.50** | **(1141.92)** ^ao^ | **3730.87** | | **(2084.41)** ^bo^ | **0.001** |
| Diptera larvae | **450.35** | **(959.42)** ^oo^ | **1904.55** | | **(2588.78)** ^oo^ |  | **150.07** | **(279.47)** ^oo^ | **936.54** | | **(1252.16)** ^oo^ | **0.217** |
| Nematocera larvae | 241.60 | (680.08) ^oo^ | 1520.16 | | (2192.94) ^oo^ |  | 2.04 | (4.56) ^oo^ | 254.25 | | (521.49) ^oo^ | 0.132 |
| Chironomidae larvae | ‒ |  | 1.06 | | (3.00) ^oo^ |  | ‒ |  | ‒ | |  | 0.522 |
| Cecidomyiidae larvae ** | 4.40 | (9.74) ^ao^ | 2.12 | | (3.21) ^ao^ |  | 1.70 | (3.80) ^ao^ | 25.46 | | (20.89) ^bo^ | 0.003 |
| Sciaridae larvae | ‒ |  | 1.45 | | (1.94) ^oo^ |  | 0.34 | (0.76) ^oo^ | 2.15 | | (2.44) ^oo^ | 0.064 |
| Mycetophilidae larvae | ‒ |  | 2.32 | | (6.50) ^oo^ |  | ‒ |  | ‒ | |  | 0.515 |
| Scatopsidae larvae | ‒ |  | 3.18 | | (9.00) ^oo^ |  | ‒ |  | 9.62 | | (21.51) ^oo^ | 0.374 |
| Tipulidae larvae | 237.20 | (670.62) ^oo^ | 1510.03 | | (2189.71) ^oo^ |  | ‒ |  | 217.02 | | (444.54) ^oo^ | 0.130 |
| Limoniidae larvae | ‒ |  | 0.02 | | (0.05) ^oo^ |  | ‒ |  | ‒ | |  | 0.522 |
| Brachycera larvae | 129.15 | (227.14) ^oo^ | 211.92 | | (203.16) ^oo^ |  | 122.09 | (234.42) ^oo^ | 535.41 | | (623.27) ^oo^ | 0.155 |
| Rhagionidae larvae | 75.14 | (184.65) ^oo^ | 186.56 | | (189.01) ^oo^ |  | 111.73 | (235.11) ^oo^ | 486.09 | | (635.33) ^oo^ | 0.156 |
| Asilidae larvae | 0.02 | (0.05) ^oo^ | ‒ | |  |  | 0.03 | (0.06) ^oo^ | ‒ | |  | **0.539** |
| Empididae larvae | 16.66 | (39.95) ^oo^ | 9.90 | | (28.01) ^oo^ |  | 5.09 | (7.26) ^oo^ | 30.84 | | (44.90) ^oo^ | 0.613 |
| Syrphidae larvae | ‒ |  | 10.08 | | (28.51) ^oo^ |  | ‒ |  | ‒ | |  | 0.522 |
| Muscidae larvae | 37.33 | (101.89) ^oo^ | 5.38 | | (7.58) ^oo^ |  | 5.23 | (6.50) ^oo^ | 18.48 | | (21.15) ^oo^ | 0.693 |
| Diptera larvae indet. | 79.60 | (106.65) ^oo^ | 172.47 | | (272.62) ^oo^ |  | 25.95 | (46.20) ^oo^ | 146.88 | | (227.14) ^oo^ | 0.529 |
| Coleoptera | **497.19** | **(642.72)** ^oo^ | **598.95** | | **(538.23)** ^oo^ |  | **349.72** | **(712.18)** ^oo^ | **740.18** | | **(1170.88)** ^oo^ | **0.858** |
| Coleoptera larvae | **1166.51** | **(2138.79)** ^oo^ | **1134.24** | | **(629.78)** ^oo^ |  | **575.22** | **(509.04)** ^oo^ | **2094.90** | | **(1640.65)** ^oo^ | **0.457** |

**Table S4: Results of the Analysis of Similarity for management (man.), treatment (treat.), and sampling year.** Calculations were performed without (untransformed) and with data transformation (fourth-root transformed) of all samples from both years combined (*ALL*) and separately (*1998*, *2012*), and of four main taxa (*4Taxa*: Lumbricidae, Chilopoda, Diplopoda, and Diptera larvae).

| **Abundance Grouping** | **Untransformed** | | | | | | **Fourth-Root Transformed** | | | | | |
| --- | --- | --- | --- | --- | --- | --- | --- | --- | --- | --- | --- | --- |
|  | **R manag. (M/P)** | **P** | **R treat. (m/a)** | **P** | **R year (98/12)** | **P** | **R manag. (M/P)** | **P** | **R treat. (m/a)** | **P** | **R year**  **(98/12)** | **P** |
| ***ALL*** | 0.091 | 0.001 | 0.388 | 0.001 | 0.263 | 0.001 | 0.116 | 0.001 | 0.399 | 0.001 | 0.343 | 0.001 |
| ***1998*** | 0.238 | 0.003 | 0.438 | 0.001 | ‒ | ‒ | 0.240 | 0.001 | 0.456 | 0.001 | ‒ | ‒ |
| ***2012*** | 0.095 | 0.002 | 0.499 | 0.001 | ‒ | ‒ | 0.099 | 0.002 | 0.469 | 0.001 | ‒ | ‒ |
| ***4Taxa*** | 0.095 | 0.001 | 0.581 | 0.001 | 0.201 | 0.001 | 0.100 | 0.001 | 0.392 | 0.001 | 0.307 | 0.001 |

**Figure S1: Latest version of an official aerial photo of the LTER area Kaserstattalm from the year 2010 with the location of the four studied sites indicated.** The figure was created using the official web-based spatial information systems “tirisMaps 2.0” from the federal state of Tyrol, Austria.


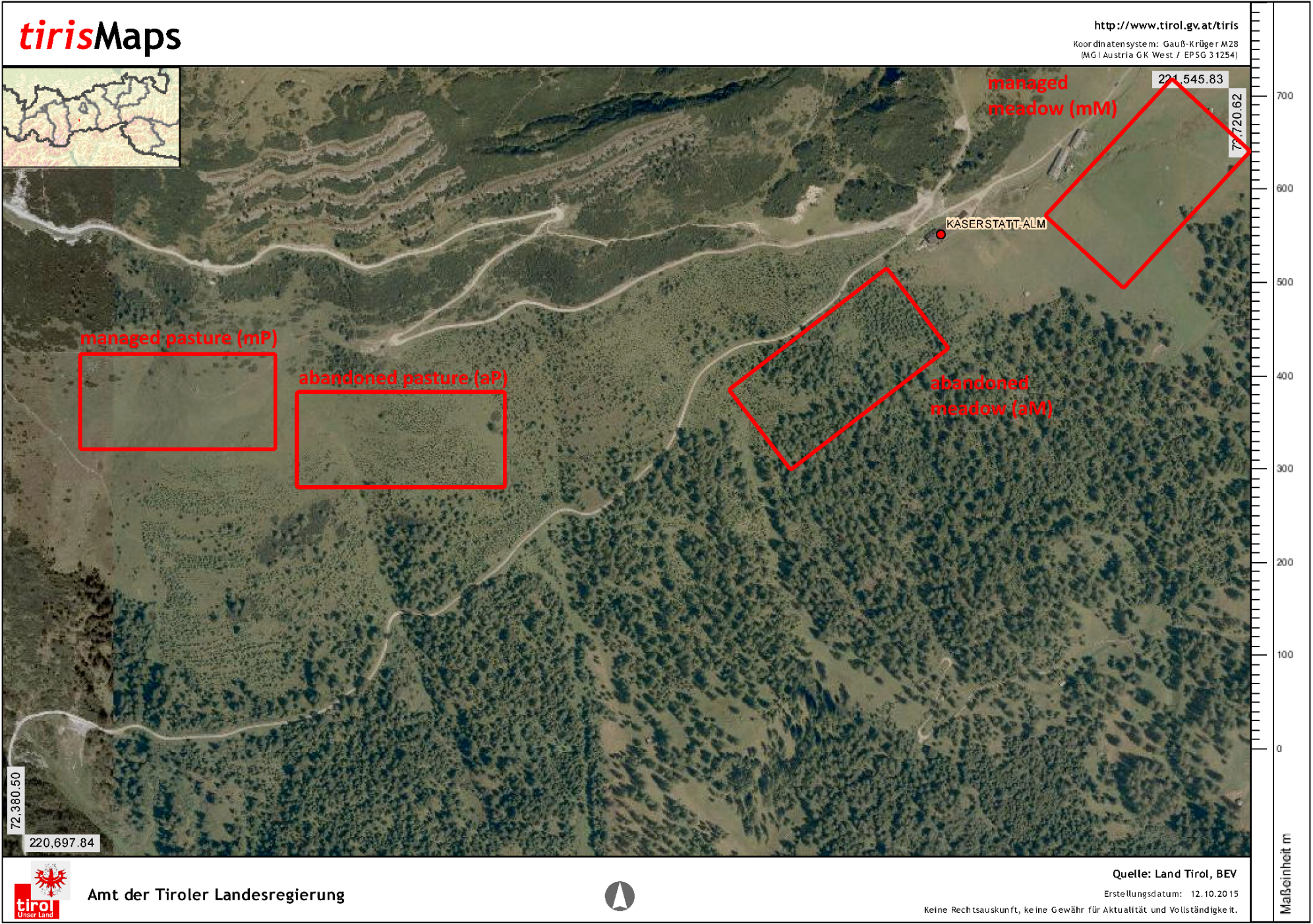


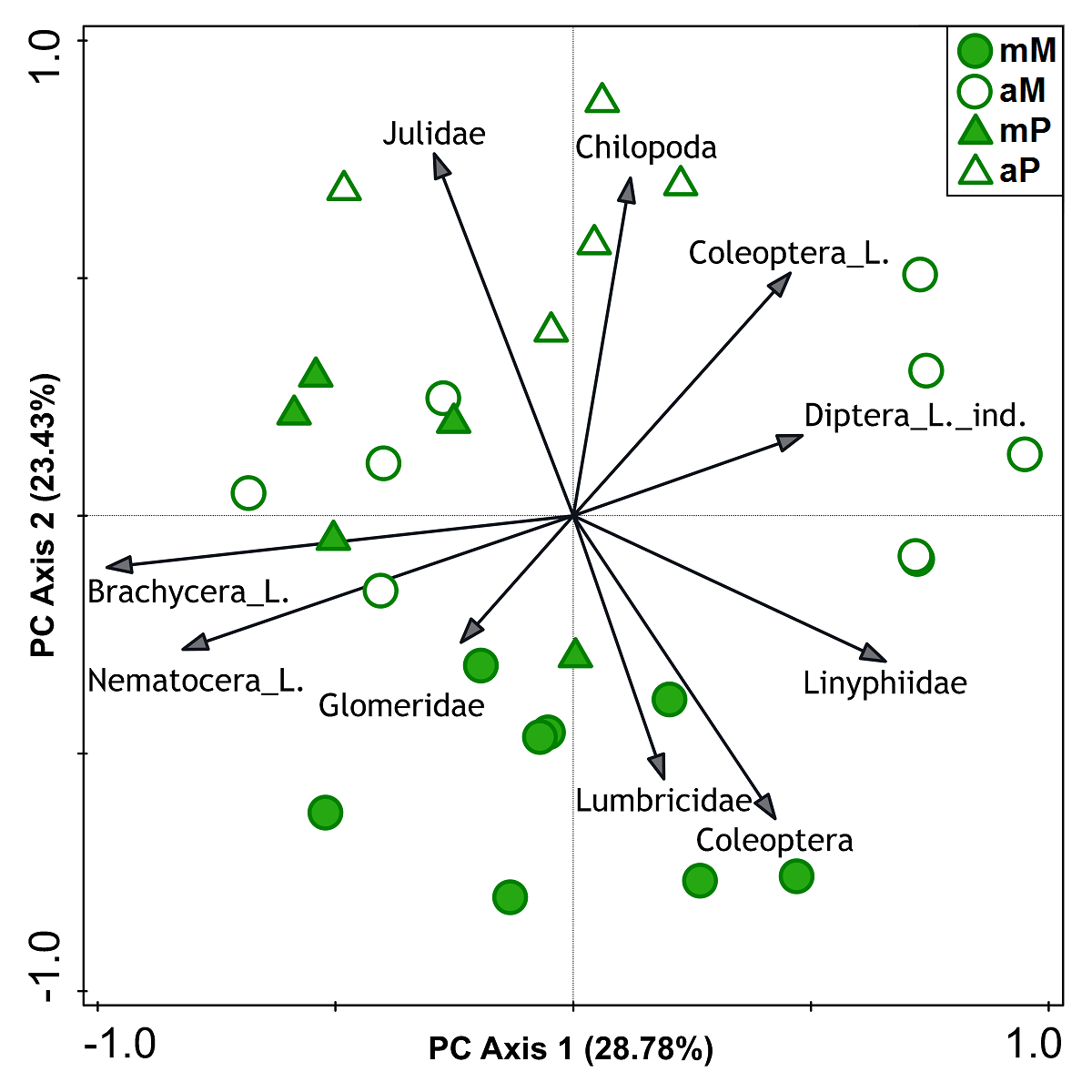


**Figure S2: Unconstrained principal component analysis (PCA) plot of log-transformed abundance (order/family level) for all 1998 samples for all four sites.** Eigenvalues axis 1: 0.2878, axis 2: 0.2343, explained variation is 52.21, total variation is 270.00


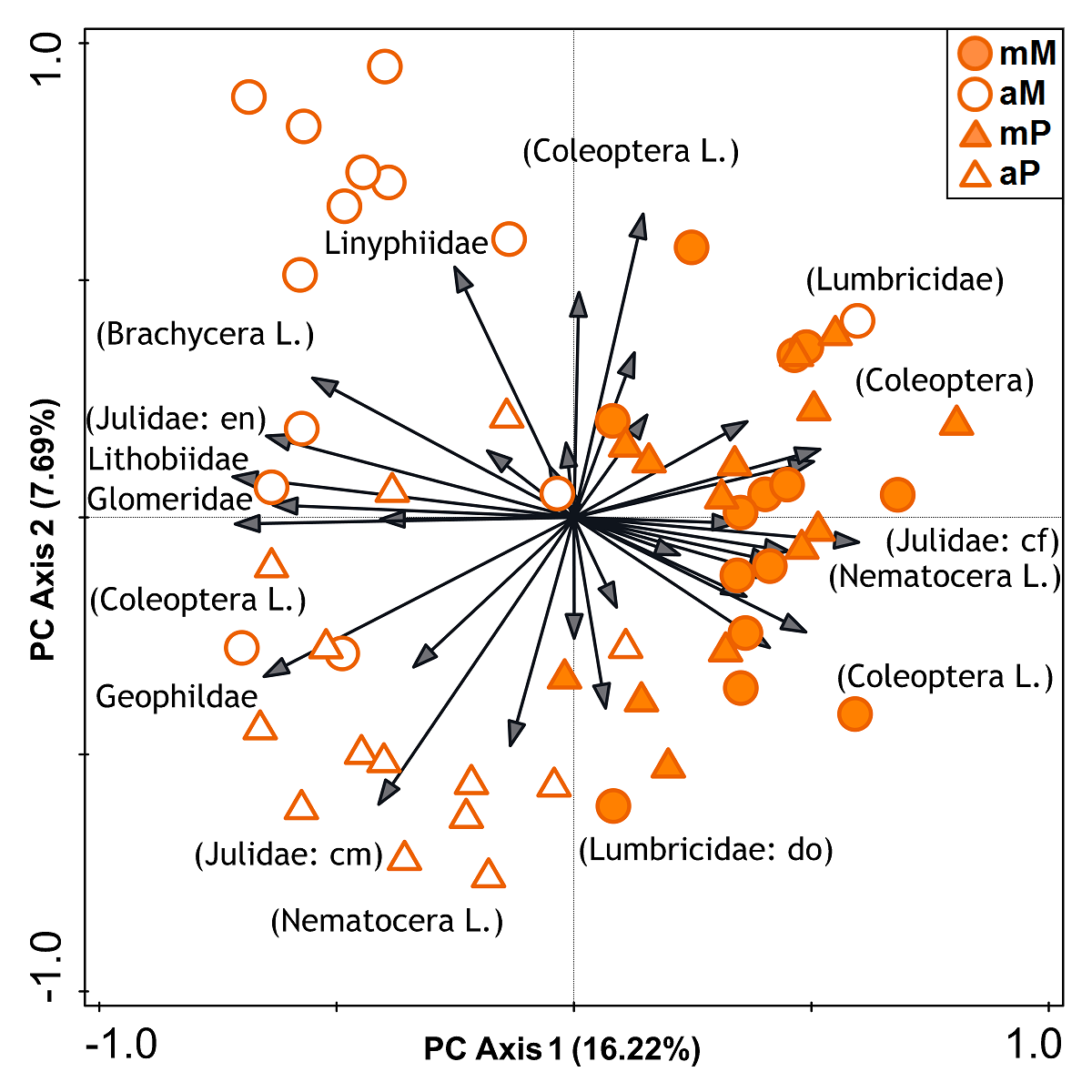


**Figure S3: Unconstrained principal component analysis (PCA) plot of log-transformed abundance at the highest taxonomic resolution (family/species level) for all 2012 samples for all four sites.** Eigenvalues axis 1: 0.1622, axis 2: 0.0769, explained variation is 23.91%, total variation is 1904.00


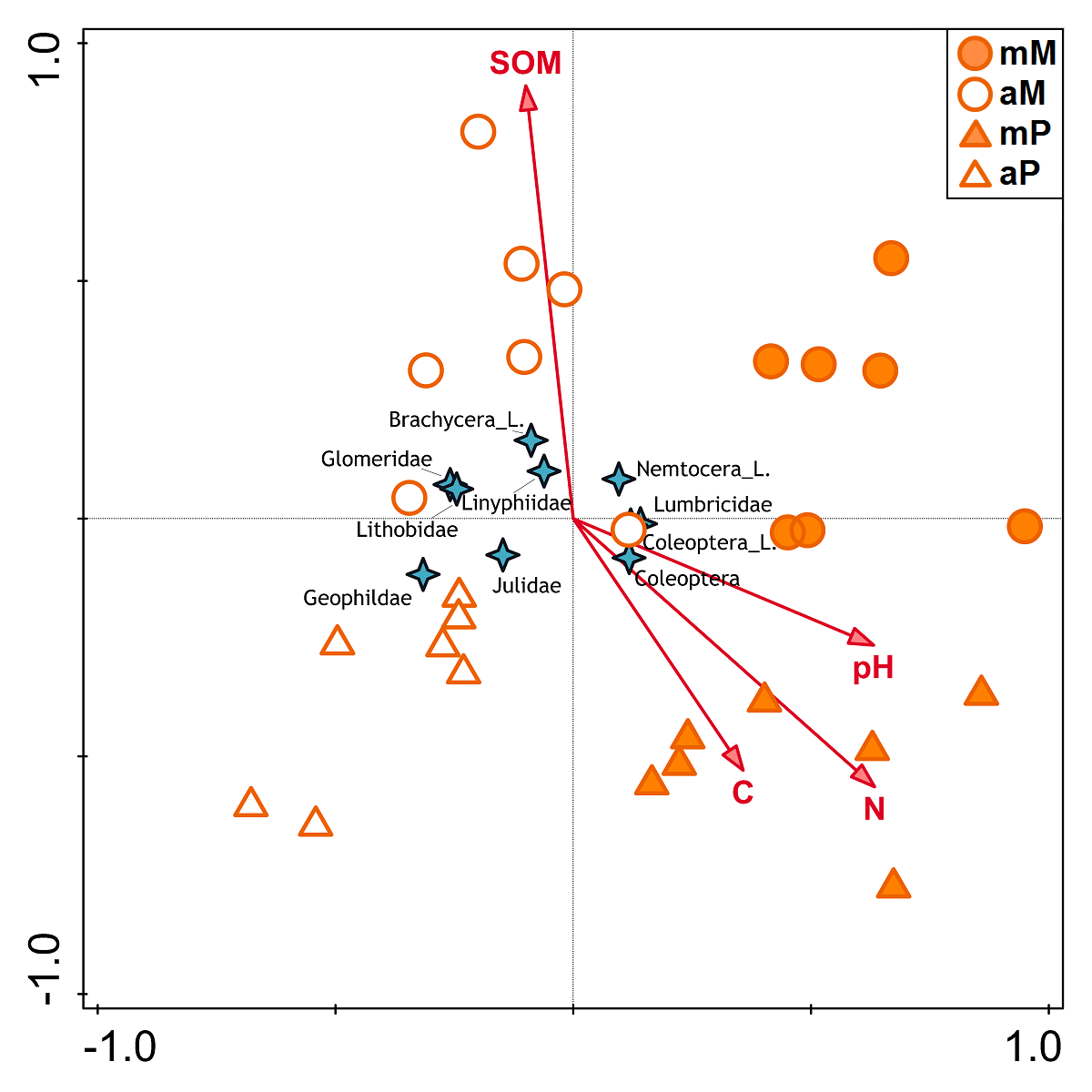


**Figure S4: Constrained canonical correspondence analysis (CCA) summarizing the variation of the abundance of soil animal groups on the four sites explained by the environmental variables for the 2012 samples.** Arrows represent soil properties (SOM, C, N, pH), while triangles represent animal groups. Eigenvalues axis 1: 0.1380, axis 2: 0.0291, test on all axes: pseudo-F = 4.4, P < 0.001.


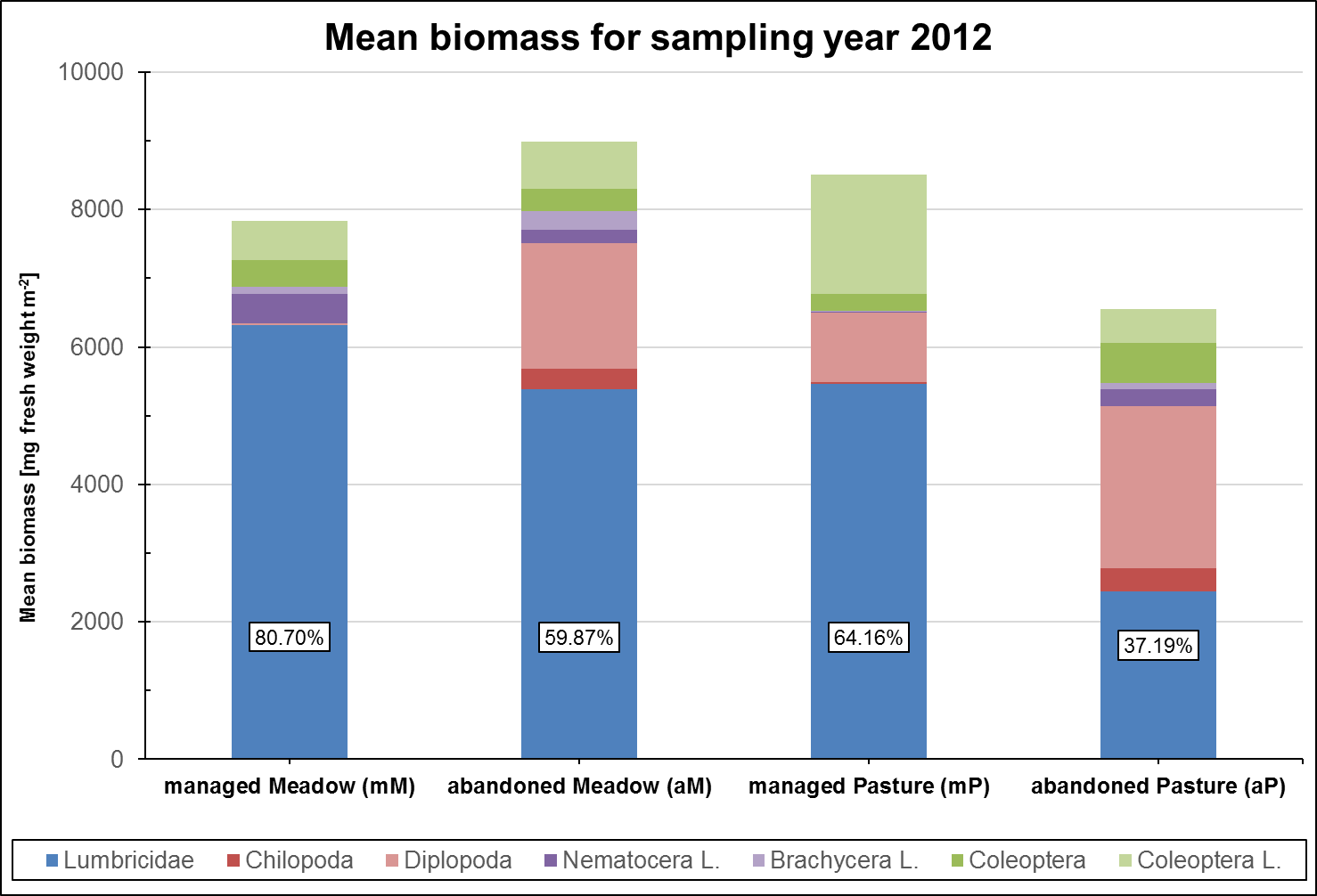


| **Biomass [mg fresh weight m^-2^]** | **managed** | | **abandoned** | | **managed** | | **abandoned** | |
| --- | --- | --- | --- | --- | --- | --- | --- | --- |
| **and percentage** | **Meadow (mM)** | | **Meadow (aM)** | | **Pasture (mP)** | | **Pasture (aP)** | |
| Lumbricidae | 6324.06 | (80.70%) | 5378.38 | (59.87%) | 5458.77 | (64.16%) | 2438.16 | (37.19%) |
| Chilopoda | – |  | 302.62 | (3.37%) | 29.21 | (0.34%) | 344.92 | (5.26%) |
| Diplopoda | 24.28 | (0.31%) | 1829.69 | (20.37%) | 1016.54 | (11.95%) | 2352.90 | (35.89%) |
| Nematocera larvae | 427.10 | (5.45%) | 196.07 | (2.18%) | 10.00 | (0.12%) | 252.11 | (3.85%) |
| Brachycera larvae | 104.55 | (1.33%) | 275.74 | (3.07%) | 10.68 | (0.13%) | 83.61 | (1.28%) |
| Coleoptera | 384.47 | (4.91%) | 324.18 | (3.61%) | 247.79 | (2.91%) | 588.04 | (8.97%) |
| Coleoptera larvae | 572.36 | (7.30%) | 676.61 | (7.53%) | 1734.91 | (20.39%) | 496.79 | (7.58%) |
| **Sum** | **7836.82** |  | **8983.27** |  | **8507.91** |  | **6556.53** |  |

**Figure S5: Mean biomass (mg fresh weight mg^-2^) of seven main groups of soil invertebrates sampled in 2012.** The figure shows the total biomasses for the four investigated sites with percentage given for Lumbricidae. The table summarizes the corresponding data with proportions of main groups per site in parentheses.


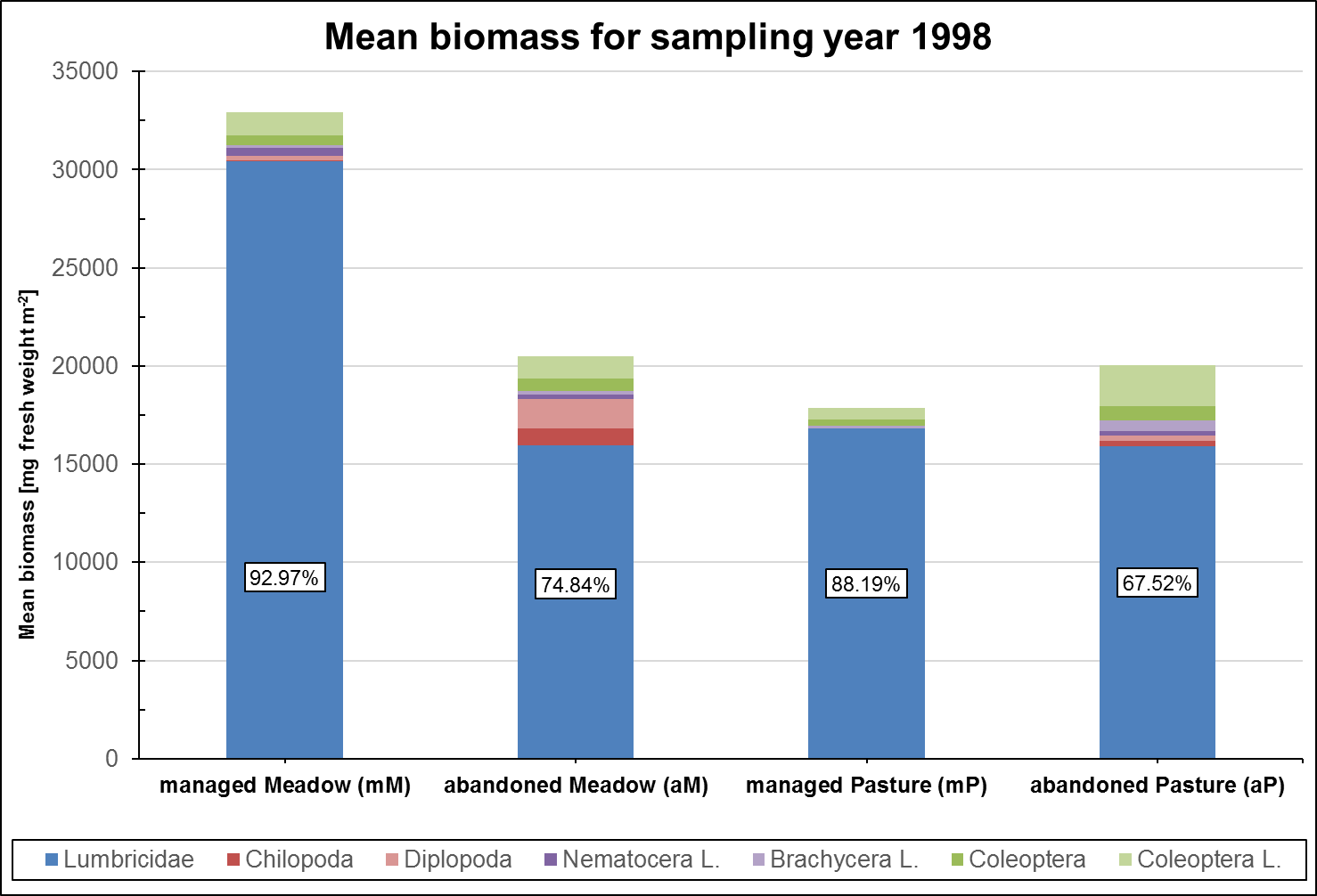


| **Biomass [mg fresh weight m^-2^]** | **managed** | | **abandoned** | | **managed** | | **abandoned** | |
| --- | --- | --- | --- | --- | --- | --- | --- | --- |
| **and percentage** | **Meadow (mM)** | | **Meadow (aM)** | | **Pasture (mP)** | | **Pasture (aP)** | |
| Lumbricidae | 30432.00 | (92.97%) | 15970.31 | (74.84%) | 16806.76 | (88.07%) | 15892.86 | (67.52%) |
| Chilopoda | 23.74 | (0.07%) | 844.94 | (3.96%) | – |  | 290.30 | (1.23%) |
| Diplopoda | 242.86 | (0.74%) | 1060.15 | (4.97%) | 1202.50 | (6.31%) | 3730.87 | (15.85%) |
| Nematocera larvae | 241.60 | (0.74%) | 1520.16 | (7.12%) | 2.04 | (0.01%) | 254.25 | (1.08%) |
| Brachycera larvae | 129.15 | (0.38%) | 211.92 | (0.99%) | 122.09 | (0.64%) | 535.41 | (2.27%) |
| Coleoptera | 497.19 | (1.52%) | 598.95 | (2.81%) | 349.72 | (1.83%) | 740.18 | (3.14%) |
| Coleoptera larvae | 1166.51 | (3.56%) | 1134.24 | (5.31%) | 575.22 | (3.02%) | 2094.90 | (8.90%) |
| **Sum** | **32733.04** |  | **21340.68** |  | **19058.33** |  | **23538.78** |  |

**Figure S6: Mean biomass (mg fresh weight mg^-2^) of six main groups of soil invertebrates sampled in 1998.** The figure shows the total biomasses for the four investigated sites with percentage given for Lumbricidae. The table summarizes the corresponding data with proportions of main groups per site in parentheses
